# Effect of Baicalin on the Proliferation of *Nosema ceranae* in *Apis cerana*

**DOI:** 10.3390/insects17050454

**Published:** 2026-04-24

**Authors:** Xu Han, Jin-Hua Xiao, Wu-Jun Jiang, Zhi-Jiang Zeng

**Affiliations:** 1 Honeybee Research Institute, Jiangxi Agricultural University, Nanchang 330045, China; hanxu68honeybee@sina.com (X.H.); jiangwj2260@163.com (W.-J.J.); 2 Department of Animal Science, Jiangxi Biotech Vocational College, Nanchang 330200, China; xjha68honeybee@163.com; 3 Apicultural Research Institute of Jiangxi Province, Nanchang 330200, China; 4 Jiangxi Province Key Laboratory of Honeybee Biology and Beekeeping, Nanchang 330045, China

**Keywords:** honeybees, *Nosema ceranae*, *Scutellaria baicalensis*, baicalin, chitinase gene

## Abstract

*Nosema ceranae* is a common and highly contagious fungal pathogen that primarily infects the gut of adult honeybees, causing nosemosis. As a chronic disease of the digestive system, it poses a global threat to honeybee health and colony sustainability. This study aimed to investigate the inhibitory effects of different concentrations of *Scutellaria baicalensis* aqueous extract on *N. ceranae* in the intestines of infected *Apis cerana* through feeding experiments. In addition, the therapeutic efficacy of its major active component, baicalin, was evaluated, and its potential molecular mechanisms of action were explored. The results showed that, compared with the control group, administration of *S. baicalensis* aqueous extract at concentrations of 1 mg/mL, 5 mg/mL, and 10 mg/mL significantly reduced midgut spore loads (*p* < 0.05). Further experiments showed that a 0.5 mg/mL baicalin sucrose solution, prepared with 0.5% (*v*/*v*) DMSO as co-solvent, exhibited optimal solubility and significantly inhibited the proliferation of spores in the honeybee midgut. Transcriptomic analysis of *A. cerana* revealed varying numbers of significantly differentially expressed genes among the baicalin-treated (HG) group, the co-solvent control (DMSO) group, and the blank control (C) group. Four candidate DEGs associated with the effects of baicalin were further identified, namely *LOC108003965, LOC108000905, LOC107996681*, and *CYP4G11*. Gene Ontology enrichment analysis showed that, in the comparison between the HG group and the C group, these DEGs were significantly enriched in six functional categories: iron ion binding, phosphoric ester hydrolase activity, heme binding, tetrapyrrole binding, hydrolase activity (acting on ester bonds), and oxidoreductase activity (acting on paired donors, with incorporation or reduction of molecular oxygen). Collectively, these results demonstrate that *S. baicalensis* aqueous extract effectively inhibits the proliferation of *N. ceranae* within the host, and its active component, baicalin, exhibits a similar inhibitory effect. The present study proposes a novel strategy in which baicalin may enhance host endogenous chitinase-related activity to target and disrupt the spore wall, offering a new perspective for the prevention and control of honeybee nosemosis.

## Simple Summary

This study evaluated the therapeutic potential of *Scutellaria baicalensis* and its active component baicalin against *Nosema ceranae* infection. The results showed that both the *S. baicalensis* aqueous extract and baicalin significantly inhibited the proliferation of this pathogen in *Apis cerana*. Transcriptomic analysis revealed that baicalin promoted the expression of the intestinal chitinase-related genes in infected *A. cerana*. This enhanced expression is considered a key mechanism that may contribute to improved host capacity for spore elimination. This study provides a theoretical basis for the application of baicalin in the prevention and treatment of *N. ceranae*, and offers a reference for the development of novel anti-*Nosema* agents.

## 1. Introduction

Honeybees are of global importance due to their large-scale cultivation, advanced husbandry techniques, and broad applications, providing essential support for ecological stability and sustainable agricultural development [1,2,3]. With the continued expansion of global crop production, honeybees, as highly efficient pollinators, have attracted significant attention for their contributions to agriculture [4,5]. However, alongside the rapid growth of the global beekeeping industry, the prevention and control of honeybee diseases have emerged as critical challenges that cannot be overlooked.

*Nosema ceranae* is an obligate intracellular fungal pathogen characterized by potent environmental adaptability [6,7,8]. This trait enables year-round infestation of hosts, posing a persistent threat to colony health and sustainability [9]. The pathogenesis of *N. ceranae* involves the specific infection of midgut epithelial cells in adult honeybees, leading to structural and functional damage and ultimately resulting in nosemosis [10]. This disease is a chronic infectious disorder of the digestive system, characterized by a prolonged incubation period and sustained detrimental effects, and represents a significant threat to both individual honeybees and colony health [11]. *N. ceranae* is transmitted primarily through the fecal–oral route via contaminated food and water, facilitating its spread within and between colonies [12]. Furthermore, evidence indicates that honeybees can also become infected during activities such as hive cleaning, trophallaxis (food exchange), and mating [13,14,15,16]. Following ingestion, spores germinate in the midgut and inject their sporoplasm into epithelial cells via the polar tube; subsequent intracellular proliferation leads to host cell rupture and the release of new spores, which then infect neighboring cells [17]. During the later stages of infection, spores are excreted in feces and released into the environment, serving as a continuous source of transmission [18].

Facilitated by global apicultural trade and the movement of honeybee colonies, this pathogen has achieved worldwide dissemination and establishment, and has successfully shifted from its original host, *Apis cerana*, to *Apis mellifera* [19,20,21,22,23,24,25]. It has now emerged as the primary causative agent of nosemosis in the latter species [26,27].

Studies have demonstrated that, compared to its original host (*A. cerana*), *N. ceranae* exhibits greater virulence in its new host *A. mellifera* [8,26]. Beyond impairing honeybee physiology and reducing lifespan, the pathogen induces energetic stress and suppresses immune and antioxidant defenses in *A. mellifera*, thereby promoting its own replication [14,28,29,30,31,32]. Therefore, this disease not only threatens the sustainable development of the global beekeeping industry but also poses a serious challenge to agricultural systems that depend on honeybee pollination, with direct implications for ecosystem stability and global food security [33,34]. Fumagillin, historically the only approved agent for the control of nosemosis in honeybees, was widely used in the global beekeeping industry for many years [18]. However, subsequent studies have confirmed its toxicity to honeybees and the potential health risks associated with residues in apiculture-derived products [35]. Consequently, its use has been prohibited in major beekeeping regions, including the European Union [36]. To date, safe, effective, and economically viable alternatives for the treatment of this disease remain lacking.

The control of *N. ceranae* remains highly challenging, owing not only to its concealed parasitic behavior and complex transmission pathways but also to the unique characteristics of its spores. The spore wall exhibits a unique structure and chemical composition, forming a robust barrier against environmental stressors and pharmacological interventions [37,38]. This allows the spores to maintain long-term infectivity while substantially reducing the efficacy of conventional control strategies.

Throughout the history of human efforts to combat disease, plant-derived medicines have played a vital role. Extensive evidence indicates that plants rich in natural antimicrobial components can be used not only directly in anti-infective therapies but also as valuable resources for developing novel antifungal agents [39]. Gajger et al. [40] found that feeding *A. mellifera* with “Nozevit” (an oak bark extract) significantly reduced *N. apis* spore loads in the midgut of infected bees. Maistrello et al. [41] demonstrated that honeybees fed thymol- or resveratrol-supplemented diets exhibited significantly lower *Nosema* infection rates, with resveratrol also extending bee longevity. Borges and colab [42] further showed that several plant-derived compounds, including sulforaphane (from cruciferous vegetables), cinnamyl alcohol (from oregano oil), and naringenin (from citrus fruits), significantly reduced *N. ceranae* spore loads in honeybees compared with infected controls. However, these compounds exhibited toxicity to honeybees at higher concentrations. The chemical complexity of plant-derived substances and the variability among extract batches remain key factors limiting their efficacy in controlling *N. ceranae* infections. Therefore, to achieve consistent therapeutic outcomes, it is essential to identify the active compounds and determine their optimal effective dosages.

This study aimed to evaluate, through feeding experiments, the inhibitory effects of the aqueous extract of *Scutellaria baicalensis* and its active component baicalin on midgut spore loads in infected honeybees. In addition, transcriptomic analysis was employed to elucidate the pharmacological effects and potential mechanisms of baicalin, thereby providing a theoretical basis for the screening of anti-*N. ceranae* bioactive compounds.

## 2. Materials and Methods

### 2.1. Fungal Spores and Honeybees

In this study, *N. ceranae* spores were obtained from the Honeybee Research Institute of Jiangxi Agricultural University and purified using the discontinuous Percoll gradient centrifugation [43]. Microscopic examination and PCR verification were performed to confirm the identity of the spores as *N. ceranae* (see Supplementary Table S1). Fresh spores were used to ensure completion of the infection process within 8 h. Sealed brood frames were collected from three healthy colonies at the same institute and placed in an incubator (35 °C, 75% humidity) to obtain newly emerged *A. cerana* workers within 24 h. Microscopic examination confirmed that the newly emerged workers were free of infection by spore-forming fungi.

### 2.2. Medicinal Plant and Bioactive Constituent

Based on previous literature, *S. baicalensis* and its active constituent baicalin, both known for their antimicrobial properties, were selected as experimental materials in this study. *S. baicalensis* (dried powder, baicalin content: 10.1%) and baicalin (analytical standard; purity ≥ 98%) were supplied by Pinxuantang Trading Firm (Bozhou, China) and Shanghai Macklin Biochemical Technology Co., Ltd. (Shanghai, China), respectively. Dimethyl sulfoxide (DMSO) (HPLC, Purity: ≥99.9%) was obtained from Shanghai Macklin Biochemical Technology Co., Ltd. (Shanghai, China). Preliminary experiments determined that the maximum safe concentration of *S. baicalensis* aqueous extract for feeding *A. cerana* infected with *N. ceranae* was 10 mg/mL (see Supplementary Figure S1). In addition, based on previous reports [44], the maximum safe concentration of dimethyl sulfoxide (DMSO) in 50% (*w*/*v*) sucrose solution for feeding was 0.5% (*v*/*v*). Accordingly, the optimal concentration of baicalin solution used in this study was established at 0.5 mg/mL.

Preparation of *S. baicalensis* extract solution: 1 g of the powder was added to 100 mL of 50% (*w*/*v*) aqueous sucrose solution and subjected to ultrasonic extraction at 60 °C for 1 h. The extract was then filtered and diluted with the same sucrose solution to obtain *S. baicalensis* extract solutions at concentrations of 10 mg/mL (high), 5 mg/mL (medium), and 1 mg/mL (low).

Preparation of baicalin solution: Due to its poor water solubility, 20 mg of baicalin was first dissolved in 200 μL of DMSO, as a co-solvent, followed by dilution with 50% (*w*/*v*) sucrose solution to obtain a final concentration of 0.5 mg/mL. A control solution containing DMSO (5 μL/mL) in 50% (*w*/*v*) sucrose solution was prepared in parallel for comparison.

### 2.3. N. ceranae Infection

Based on the spore counts, an appropriate volume of 50% (*w*/*v*) sucrose solution was added to a known quantity of spores to prepare a suspension at a concentration of 5 × 10^4^ spores/μ L. Each newly emerged worker honeybee (within 24 h post-eclosion) was orally inoculated with 2 μL of the spore suspension, corresponding to an infection dose of 1 × 10^5^ spores per honeybee. To improve feeding efficiency, all experimental honeybees were starved for 2 h in a constant-temperature incubator prior to inoculation.

### 2.4. Experimental Groups and Sample Collection

*S. baicalensis* extract experiment: four experimental groups were established, each comprising 60 honeybees with three biological replicates, for a total of 240 individuals. The feeding regimens were as follows: the control group (C) received 50% (*w*/*v*) sucrose solution, while the treatment groups (high, medium, and low concentrations) were provided with 50% (*w*/*v*) sucrose solution supplemented with *S. baicalensis* extract at concentrations of 10 mg/mL, 5 mg/mL, and 1 mg/mL, respectively. The experiment was conducted over 7 days, during which all diets were replaced every 24 h under feeding conditions. At 7 days post-infection (dpi), midgut tissues of individual bees in each group were collected separately for *Nosema* spore quantification and subsequent analyses.

Baicalin experiment: three experimental groups were established, each comprising 60 honeybees with three biological replicates, for a total of 180 individuals. The feeding regimens were as follows: the control group (C) received 50% sucrose solution; the baicalin-treated group (HG) was administered 50% sucrose solution containing 0.5 mg/mL baicalin (with a DMSO at 5 μL/mL); and the DMSO group (co-solvent control) was provided with 50% sucrose solution containing 5 μL/mL DMSO. The experiment was conducted over 7 days, with diets replaced every 24 h under feeding conditions. At 7 dpi, five worker bees were randomly selected from each replicate for midgut collection and total RNA extraction for transcriptome sequencing. The remaining bees were individually dissected, and their midgut tissues were collected for spore counting and subsequent analyses.

### 2.5. Spore Quantification and Analysis

Midgut spores from individual honeybees in each group were purified using discontinuous Percoll gradient centrifugation, and the number of mature spores was quantified using a hemocytometer. Statistical analyses were conducted using SPSS 26.0. Data conforming to a normal distribution were evaluated by one-way analysis of variance (ANOVA), while non-normally distributed data were analyzed using the Kruskal–Wallis test.

### 2.6. RNA-Seq

Total RNA was first extracted from the midgut tissues of infected honeybees in each group using a commercial RNA extraction kit (Beijing TransGen Biotech Co., Ltd., Beijing, China), following the manufacturer’s instructions. Transcriptome sequencing was then performed according to previously described methods [45]. To exclude potential interference from the co-solvent (DMSO) and to accurately identify differentially expressed genes (DEGs) associated with baicalin treatment, pairwise comparisons of gene expression levels were performed for both the *A. cerana* genome and *N. ceranae* genes in the honeybee midgut tissues across the different experimental groups, as previously described [45]. Candidate genes were selected based on the following criteria: (1) significant differential expression between the HG and C groups; (2) significant differential expression between the HG and DMSO groups; and (3) no significant differential expression between the DMSO and C groups.

Clean reads were first aligned to the reference genome of *A. cerana* (assembly version ASM1110058v1) to obtain host-derived data. Unmapped reads were further aligned to the *N. ceranae* reference genome (assembly version Ncer 3.0) to obtain microsporidian-derived data. Gene expression levels were compared across experimental groups, and DEGs were identified using the edgeR package (v3.24.3). The DEGs were then subjected to Gene Ontology (GO) and Kyoto Encyclopedia of Genes and Genomes (KEGG) enrichment analyses. The screening criteria were set as |log2FoldChange| > 1 and adjusted *p* < 0.05.

### 2.7. Validation of DEGs by RT-qPCR

To validate the reliability of the identified DEGs, RT-qPCR was performed to assess the expression of the *LOC108000905* gene, using *β-actin* as the internal reference gene (primer sequences are provided in Table S1). The PCR program consisted of an initial denaturation at 95 °C for 1 min, followed by 40 cycles of 95 °C for 15 s, 55 °C for 30 s, and 72 °C for 45 s. Relative gene expression levels were calculated using the 2^−ΔΔCt^ method. All experiments were conducted with three independent biological replicates, each containing three technical replicates.

## 3. Results

### 3.1. Effect of S. baicalensis Extract on Spore Load in Honeybees

**Figure 1:**
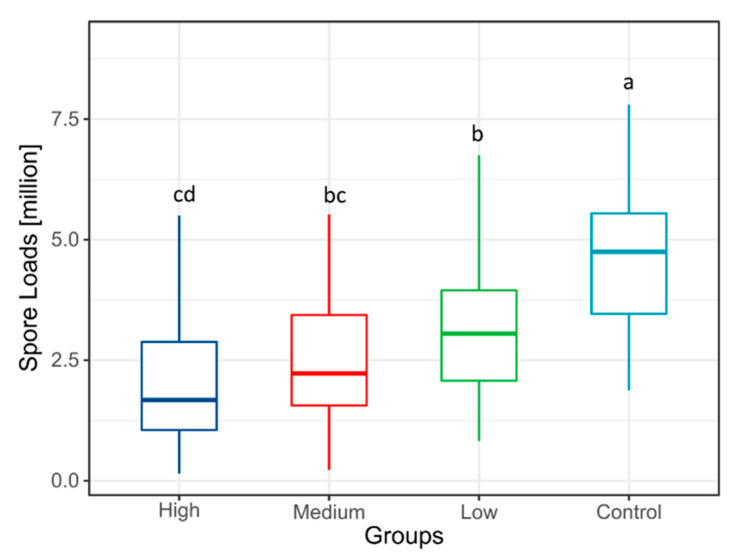
The box plot shows the spore load of *N. ceranae* in *Apis cerana* from each group under the treatment of *S. baicalensis* extract. Different letters indicate statistically significant differences between groups (*p* < 0.05).

The Kruskal–Wallis test indicated a significant difference among groups (n = 240, H = 96.190, *p* < 0.001). This indicates that at 7 dpi following the feeding experiment, there were significant differences in intestinal spore levels among different *A. cerana* groups. Pairwise comparisons further revealed significant differences in spore counts between all *S. baicalensis* groups (high, medium, and low) and the control group (Figure 1, *p* < 0.05). Detailed spore count data for each group are provided in Supplementary Table S2.

### 3.2. Effect of Baicalin on Spore Load in Honeybees

**Figure 2:**
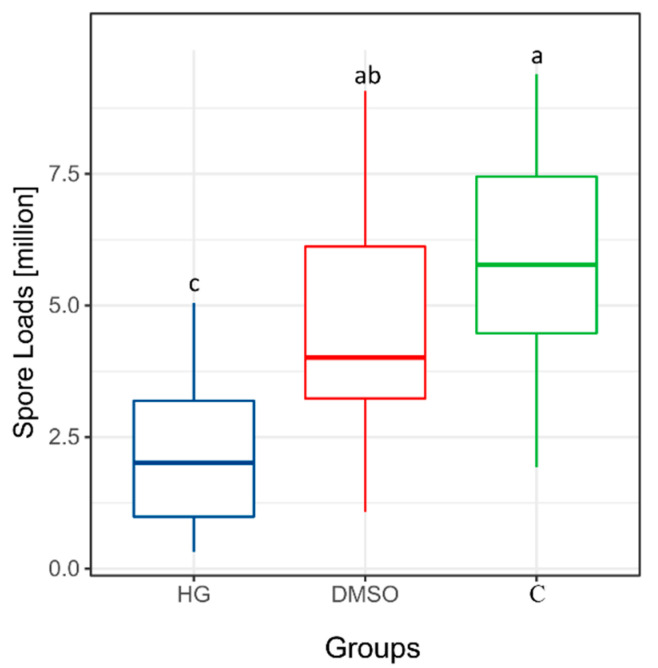
The box plot shows the spore load of *N. ceranae* in *Apis cerana* from each group under the treatment of baicalin. Different letters indicate statistically significant differences between groups (*p* < 0.05).

At 7 dpi, the Kruskal–Wallis test indicated significant differences in spore levels among groups (n = 122 after exclusion of invalid data, H = 32.057, *p* < 0.001). The HG group (baicalin-treated group) exhibited the lowest spore counts, with statistically significant differences compared to both the DMSO group (solvent control) and the C group (blank control) (*p* < 0.05). In addition, the spore count in the DMSO group was lower than that in the C group; however, this difference was not statistically significant (Figure 2, *p* > 0.05). Detailed spore count data for each group in the baicalin extract experiment are provided in Supplementary Table S3.

### 3.3. Effects of Baicalin on Host and Parasite mRNA Levels

**Figure 3:**
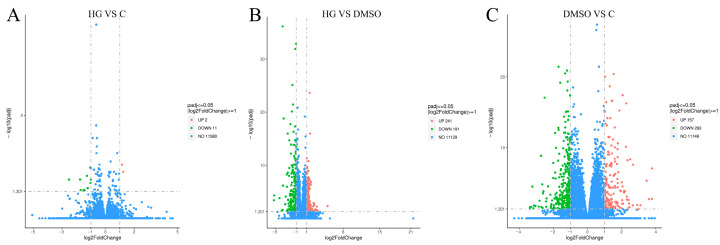
The volcano plot shows the comparison results of *Apis cerana* genes between groups. (**A**) The volcano plot shows the expression levels of DEGs between the HG group and the C group. (**B**) The volcano plot shows the expression levels of DEGs between the HG group and the DMSO group. (**C**) The volcano plot shows the expression levels of DEGs between the DMSO group and the C group.

**Figure 4:**
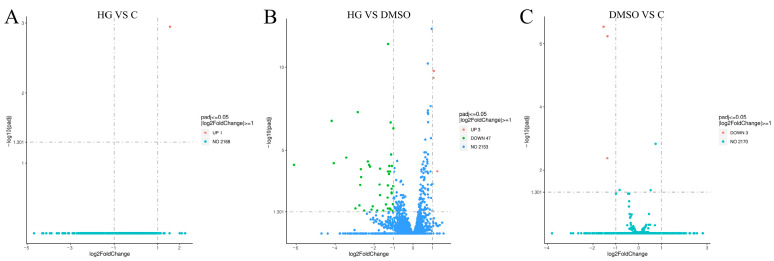
The volcano plot shows the comparison results of *N. cerana* genes between groups. (**A**) The volcano plot shows the expression levels of DEGs between the HG group and the C group. (**B**) The volcano plot shows the expression levels of DEGs between the HG group and the DMSO group. (**C**) The volcano plot shows the expression levels of DEGs between the DMSO group and the C group.

Transcriptome analysis of *A. cerana* identified 13, 432, and 450 DEGs in the comparisons of the HG group vs. C group (Figure 3A), HG group vs. DMSO group (Figure 3B), and DMSO group vs. C group (Figure 3C), respectively. Similarly, transcriptome analysis of *N. ceranae* transcriptome identified 1, 50, and 3 significantly differentially expressed genes in the comparisons of HG group vs. C group, HG group vs. DMSO group, and DMSO group vs. C group, respectively (Figure 4).

**Figure 5:**
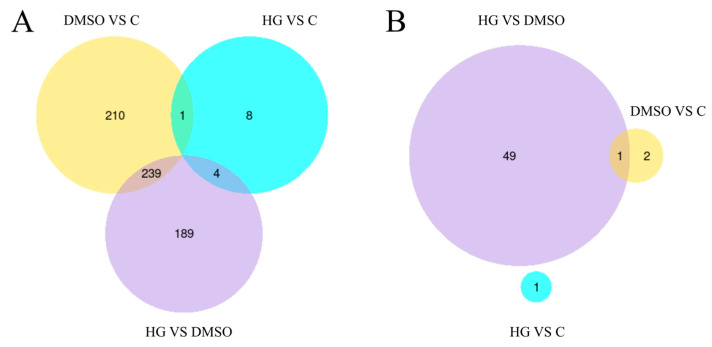
The Venn diagrams show the logical relationships among DEGs across groups. (**A**) The figure shows the Venn analysis of DEGs in *Apis cerana* among three comparison groups: HG vs. DMSO, HG vs. C, and DMSO vs. C. (**B**) The figure shows the Venn analysis of DEGs in *N. ceranae* among three comparison groups: HG vs. DMSO, HG vs. C, and DMSO vs. C. Different colors indicate different comparison combinations.

Figure 5A presents the Venn diagram analysis results for the *A. cerana* genome. Four genes met the selection criteria: *LOC108003965, LOC108000905, LOC107996681*, and *CYP4G11*. Figure 5B shows the corresponding Venn diagram analysis results for the *N. ceranae* genome, in which no differentially expressed genes satisfied the defined criteria.

**Figure 6:**
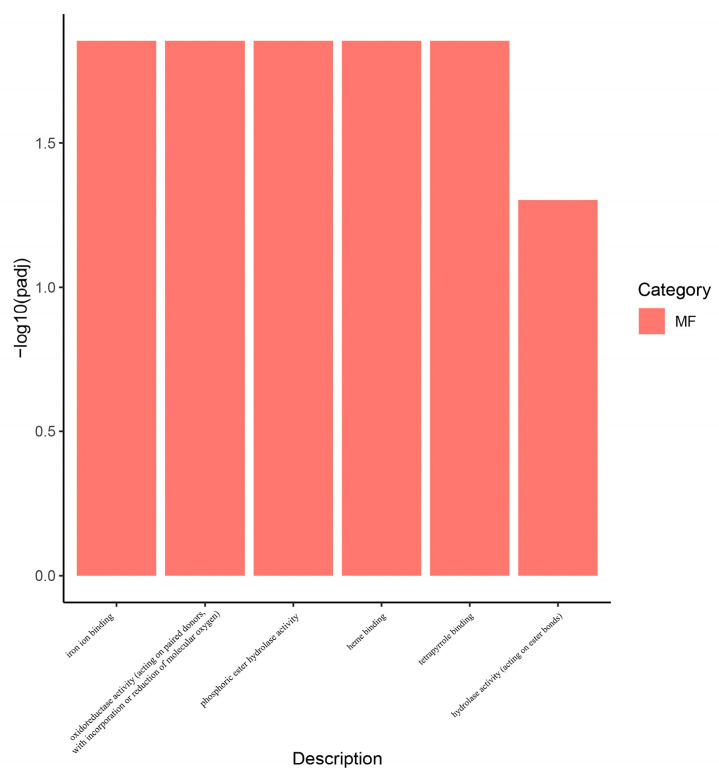
The figure shows the GO analysis plot of DEGs in *Apis cerana* (HG group vs. C group) related to the effect of baicalin treatment. MF stands for molecular function.

Based on GO and KEGG enrichment analyses of the baicalin-treated group versus the blank control group (HG group vs. C group), we further characterized the functional pathways associated with DEGs in *A. cerana*. GO enrichment analysis showed that, in the HG vs. C group, DEGs were significantly enriched in six functional categories, including iron ion binding, phosphoric ester hydrolase activity, heme binding, tetrapyrrole binding, hydrolase activity acting on ester bonds, and oxidoreductase activity acting on paired donors with incorporation or reduction of molecular oxygen (Figure 6, padj < 0.05, |log2FoldChange| > 1). In contrast, KEGG enrichment analysis showed no significantly enriched pathways in the HG vs. C group comparison.

### 3.4. qRT-PCR Validation of Key DEGs

**Figure 7:**
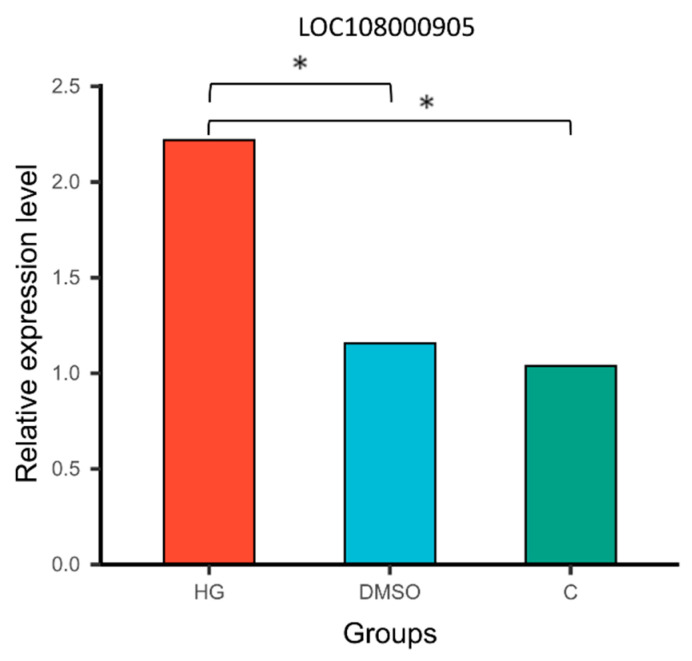
The bar plot shows the relative expression levels of LOC108000905 in the midguts of *Apis cerana* from each group. Bars with asterisk symbol indicate statistically significant differences (*p* < 0.05).

To validate the differential expression of genes among groups, the chitinase-related DEG (*LOC108000905*) was selected as a representative target. Its expression levels in the midgut of honeybees at 7 days post-infection were quantified using qRT-PCR (see Supplementary Table S5). The qRT-PCR results (Figure 7) demonstrated that *LOC108000905* expression was significantly upregulated in the HG group relative to both the C and DMSO groups (*p* < 0.05), whereas no significant difference was observed between the C and DMSO groups (*p* > 0.05). These findings were consistent with the transcriptome analysis. Primer sequences used for qRT-PCR analysis are provided in Supplementary Table S4, and the corresponding qPCR data for *LOC108000905* are presented in Supplementary Table S5.

## 4. Discussion

According to Taillon and Andreasen [46], a wide range of compounds, including herbal extracts, can be classified as potential nutraceuticals. These compounds exert their effects through diverse mechanisms to prevent and treat various diseases. Among them, one class comprises compounds with direct antimicrobial activity, which can be used to control *N. ceranae* infection [47]. These agents may either directly inactivate *N. ceranae* spores or inhibit their growth and development. Another class includes compounds with anti-inflammatory properties, which are also considered promising for controlling *N. ceranae* infection [42]. Although these compounds may not directly eliminate spores, their anti-inflammatory effects may alleviate symptoms of *N. ceranae* infection and enhance the overall health and longevity of infected honeybees. In the present study, administration of *S. baicalensis* extract at 7 dpi significantly reduced midgut spore loads of honeybees across all treatment groups compared with the blank control group (*p* < 0.05). Among the tested concentrations, 10 mg/mL was identified as the optimal dose, as it exhibited the strongest inhibitory effect on *N. ceranae* while remaining safe for *A. cerana* feeding, based on our preliminary experimental results. The upper limit of the optimal concentration range may be further refined through evaluation of higher concentrations (see Supplementary Figure S1). Additionally, the results of this study showed that baicalin, major bioactive component of *S. baicalensis,* significantly reduced the midgut spore loads in honeybees at a concentration of 0.5 mg/mL, thereby playing an important role in inhibiting *N. ceranae* infection.

Transcriptomic analysis revealed significant differential expression of genes for both *A. cerana* and *N. ceranae* across the experimental groups. Based on previous research experience, we further identified significant DEGs associated with the effects of baicalin using Venn diagram analysis, and selected these as candidate genes for further investigation. Ho et al. [48] reported that DMSO exerts a protective effect on *A. mellifera*, improving the survival of infected individuals by enhancing their adaptability or ability to cope with infection, while having no effect on the proliferation of *N. ceranae*. The authors suggested that DMSO treatment primarily influences gene expression levels in *A. mellifera*, rather than significantly altering global biochemical pathways or related regulatory modules. This finding is consistent with our results. The screening results identified four significantly differentially expressed honeybee genes associated with the effects of baicalin: *LOC108003965, LOC108000905, LOC107996681*, and *CYP4G11*. No corresponding differentially expressed genes were detected in *N. ceranae*. This may be attributable to the presence of the *N. ceranae* spore wall, which could act as a physical barrier that limits drug entry, thereby preventing baicalin from exerting a direct effect and resulting in the absence of detectable transcriptomic changes. Based on these findings, we further analyzed the pathways enriched by baicalin-associated *A. cerana* DEGs in the comparison between the baicalin-treated group and the blank control group (HG vs. C). GO enrichment analysis revealed that these DEGs were significantly enriched in six functional categories: iron ion binding, phosphoric ester hydrolase activity, heme binding, tetrapyrrole binding, hydrolase activity (acting on ester bonds), and oxidoreductase activity (acting on paired donors, with incorporation or reduction of molecular oxygen). In contrast, KEGG enrichment analysis did not identify any significant enriched pathways. This suggests that baicalin may exert its anti-microsporidian effects through mechanisms not captured by KEGG pathway annotations.

Transcriptomic analysis revealed that although *LOC108000905* (a DEG) in *A. cerana* lacks functional annotation, its homologs, such as *LOC551600* (*Cht5*) in *A. mellifera* (see Supplementary Figure S2) exhibit chitinase activity, suggesting that *LOC108000905* may also function as a chitinase. Holt et al. [49,50,51] reported that the seminal fluid of *A. mellifera* contains antimicrobial components, including chitinase, which degrades chitin, a major structural component of insect exoskeletons and fungal spore cell walls, and is therefore considered a potential factor in the neutralization of *N. apis* spores. Furthermore, Peng et al. [52] demonstrated that seminal fluid chitinase can weaken and potentially disrupt the chitin-based cell wall of *N. apis* spores, thereby interfering with the spore life cycle and potentially inducing premature germination. As early as 2016, Grassl et al. [53] identified *Cht5* in honeybee seminal fluid. Subsequently, Houdelet et al. [53,54] reported the presence of *Cht5* in the honeybee gut, confirming that this enzyme is not restricted to the seminal fluid. Doublet et al. [55] further demonstrated that the expression of the chitinase-related gene *Cht5* in *A. mellifera* is upregulated following *Nosema* infection, suggesting a potential role in host defense. Consistently, Holt et al. [49] proposed that chitinase is produced in the gut of infected *A. mellifera,* where it mediates a localized antifungal response against ingested *N. apis* spores. Altogether, these findings are consistent with our results, showing that baicalin-treated *A. cerana* exhibited significant upregulation of the *Cht5* homolog *LOC108000905*, accompanied by a marked decrease in midgut *Nosema* spore load.

Chitin is a primary component of fungal cell walls and plays a critical role in maintaining cell morphology and integrity, as well as mediating key processes during host infection. Chitinases have been widely explored as biological control agents in both medical and agricultural contexts due to their ability to degrade chitin. Previous studies have shown that certain bacterial-derived chitinases can effectively inhibit fungal growth by hydrolyzing chitin in the cell wall, thereby contributing to the control of various fungal diseases [56]. Therefore, chitinase is considered a promising agent for the biological control of fungal pathogens. In this study, we also observed that the expression of *CYP4G11* was significantly downregulated in the midguts of honeybees, concomitant with a reduction in spore counts. Previous studies have shown that *CYP4G11* is responsive to oxidative stress and may play a role in protecting honeybees against oxidative damage [57]. A plausible explanation for this phenomenon is that the honeybee midgut serves as the primary site of invasion by *N. ceranae* spores. As the spore load decreases, associated stress factors, such as oxidative stress, are alleviated, leading to reduced expression of *CYP4G11*. Based on these findings, we propose that baicalin may upregulate the expression of the chitinase homolog *LOC108000905* in the intestines of infected honeybees, thereby promoting chitinase production. This, in turn, inhibits the activity and proliferation of *N. ceranae* spores through degradation of chitin in the spore cell wall. Although several natural products, such as thymol and resveratrol, have been reported to exhibit inhibitory effects against *Nosema*, baicalin at therapeutic concentrations demonstrates a more favorable safety profile for honeybees, with no observable host mortality [40,41,42].

The above findings suggest that baicalin may promote chitinase-mediated degradation of the spore wall. However, as an obligate intracellular pathogen [17], *N. ceranae* mainly reproduces within host cells, and the chitin degradation of the spore wall is most likely to occur before host cell invasion or during the formation of new spores. Therefore, we propose that baicalin exerts two main effects in the honeybee gut: a preventive effect by inhibiting spore germination, and a suppressive effect by reducing overall spore burden. In future studies, we will focus on the practical application of baicalin. This includes enhancing its physicochemical properties, such as improving water solubility and bioavailability through salt formation; systematically evaluating its cost-effectiveness, solution stability (e.g., temperature, pH, and storage-related degradation), and field efficacy against *N. ceranae*; and assessing residue levels and safety in honeybees to ensure the quality and safety of apiculture products.

Overall, these findings provide a theoretical basis for the development and standardized production of anti-*Nosema* pharmaceuticals, and also highlight the potential of novel enzyme-based strategies targeting spore wall degradation.

## 5. Conclusions

This study demonstrated for the first time that *S. baicalensis* and its active component baicalin significantly inhibit the proliferation of *N. ceranae* in infected honeybees (*A. cerana*). Furthermore, transcriptomic analysis revealed that baicalin significantly upregulates the expression of the chitinase homologous gene in the host, suggesting its potential as a biological control strategy against *N. ceranae* infection.

## Data Availability

The data presented in this study are openly available in Transcriptome data at http://www.ncbi.nlm.nih.gov/bioproject/1443012, https://www.ncbi.nlm.nih.gov/bioproject/1445512, reference number PRJNA1443012; PRJNA1445512.

## Supplementary Materials

The following supporting information can be downloaded at: https://www.mdpi.com/article/10.3390/insects17050454/s1, Figure S1: Survival curves of baicalin-fed honeybees over 7 days. Figure S2: Diagram of homology analysis of the target gene LOC108000905. Table S1: Microscopic view and PCR verification of *N. ceranae* spores. Table S2: *Scutellaria baicalensis* extract experimental spores data. Table S3: Baicalin experimental spores data. Table S4: Primers used for qRT-PCR and their sequences. Table S5: qPCR data of LOC108000905.
