# Supplementary figures and images for: Effect of Baicalin on the Proliferation of Nosema ceranae in Apis cerana
Source: Insects. 2026 Apr 24;17(5):454. doi: 10.3390/insects17050454 (PMC13206831; doi:10.3390/insects17050454)

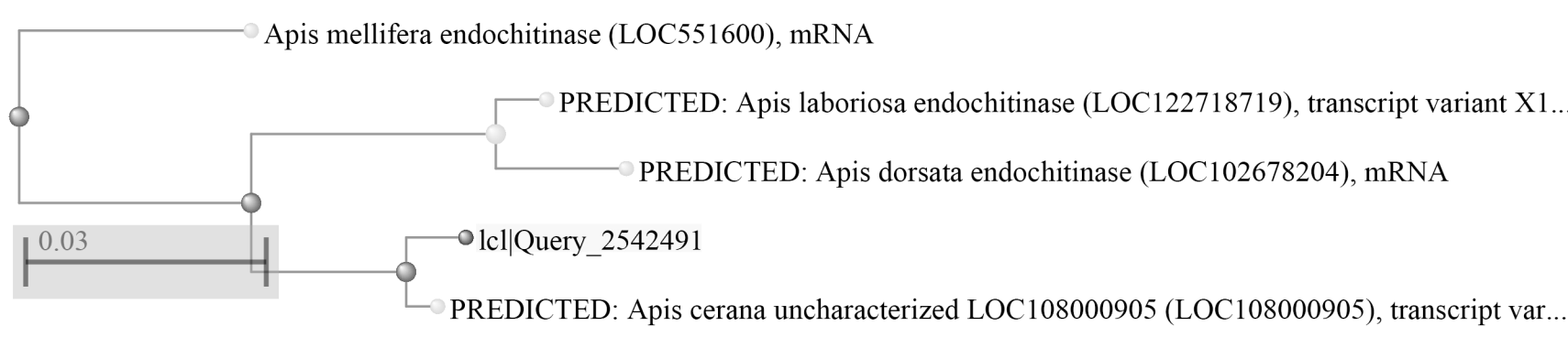

Supplement: Supplementary file 1 [file insects-17-00454-s001.zip › Supplementary Figure S2.pdf]

Survival curves of baicalin-fed honeybees over 7 days.

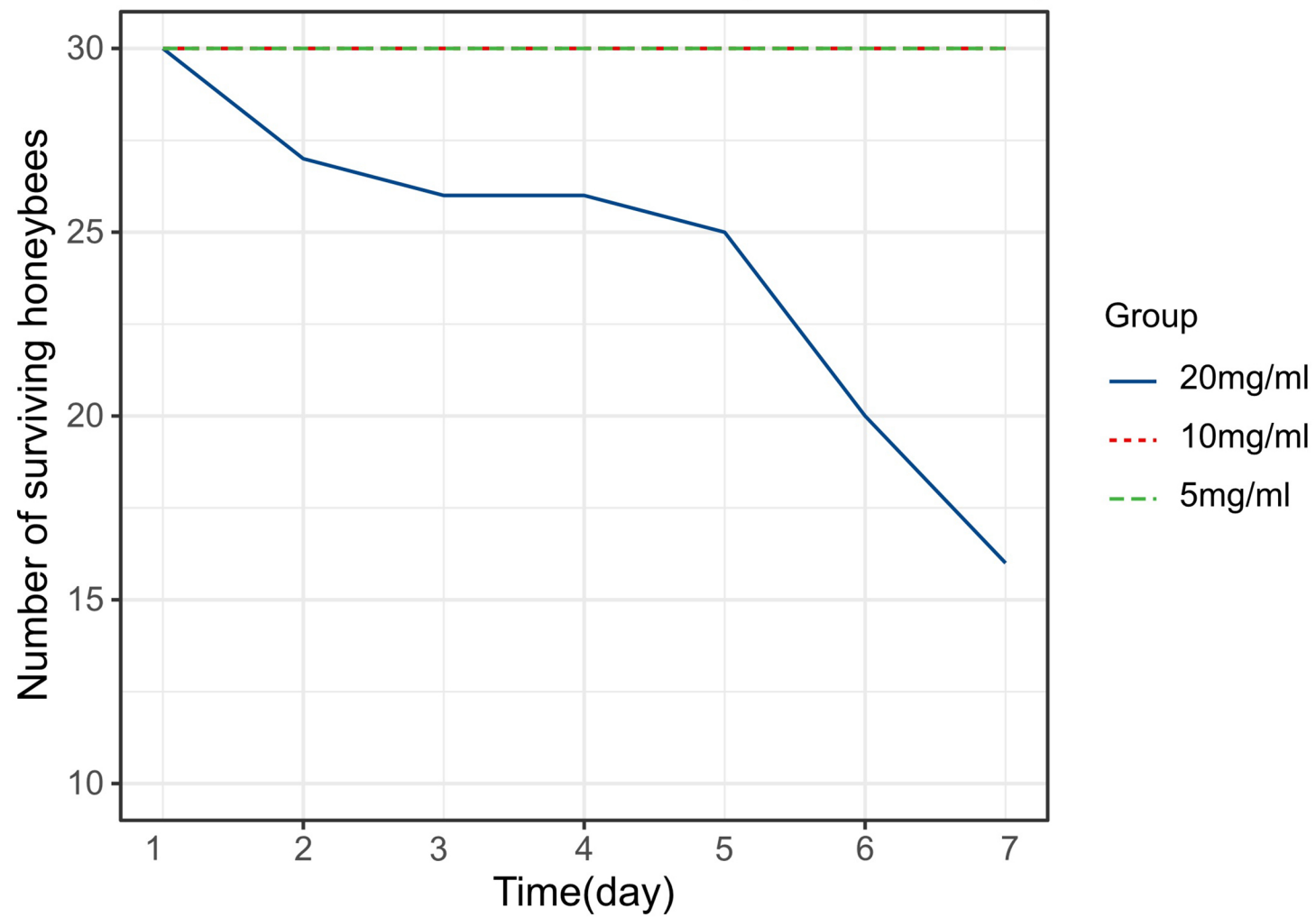

Supplement: Supplementary file 1 [file insects-17-00454-s001.zip › Supplementary Figure S1.pdf]
